# Data on terpene content in pre-rolled cone paper infused with terpene-containing flavours based on the formation of a partially soluble precipitate

**DOI:** 10.1016/j.dib.2024.110623

**Published:** 2024-06-10

**Authors:** Arthur Karangan, Sherwin Wardhana Rahardjo, Antonius Jimmy Widagdo, Shella Permatasari Santoso, Suryadi Ismadji

**Affiliations:** a Research and Development Department, PT. Mitra Prodin, Jl. Prof. Dr. Ida Bagus Mantra, Ketewel, Kecamatan Sukawati, Kabupaten Gianyar, Bali 80582, Indonesia; b Chemical Engineering Department, Faculty of Engineering, Widya Mandala Surabaya Catholic University, Jl. Kalijudan No. 37, Surabaya 60114, East Java, Indonesia; c Collaborative Research Center for Sustainable and Zero Waste Industries, Kalijudan 37, Surabaya 60114, East Java, Indonesia

**Keywords:** Terpene-infused cone, Pre-rolled cone, Reddish brown precipitate, Terpene glue, Degradation rate, UV-Vis spectrophotometer

## Abstract

The high volatility of the terpenes contained in flavour-containing terpene (FCT) products causes the loss of these contents during product storage; thus, measuring the loss of FCT content during storage is important to estimate the final content. This work provides data on the reduction in FCT content of infused pre-rolled paper cones after 1 to 7 days of storage. Determination of FCT content was based on the formation of a reddish-brown precipitate resulting from the reaction of terpene moiety in FCT with sulphuric acid. Then, the absorbance of the precipitate was analysed using the UV-Vis Spectrophotometric method at a visible wavelength of 538 nm. A calibration standard curve was prepared concerning the concentration of the original FCT sample and used to determine the FCT content in infused pre-rolled paper. The FCT content on the first day of storage decreased and increased again after seven days of storage due to condensation. The data on the FCT content reduction as the effect of additive added was also evaluated.

Specifications Table

**Table:** 

Subject	Analytical Chemistry: Spectroscopy
Specific subject area	Practical estimation of terpene content*.*
Type of data	Table and Figures. Raw and Analysed data.
Data collection	Absorbance measurement at 538 nm using UV-Vis spectrophotometer (Genesys 150, Thermo Fisher Scientific, USA)
Data source location	Flavour-containing terpene (FCT), pre-rolled paper, and glue are products of PT. Mitra Prodin (Gianyar, Bali, Indonesia) Data collection performed at: Institution: Widya Mandala Surabaya Catholic University City: Surabaya Country: Indonesia
Data accessibility	Repository name: Data on terpene content in pre-rolled cone paper infused with terpene-containing flavours based on the formation of a partially soluble precipitate Data identification number: 10.17632/h5nyfd3y92.2 Direct URL to data: https://data.mendeley.com/datasets/h5nyfd3y92/2
Related research article	None

## 1 Value of the Data

• This paper presents a simple, robust, and highly practicable method for estimating the amount of terpene-containing product based on the formation of a reddish brown precipitate that has a linear response using spectrophotometric measurement at 538 nm.
• The data on FCT decrement as the effect of operating conditions would provide insight for the industrial sector into selecting the appropriate method for storing the terpene-infused pre-rolled cone products.
• It is anticipated that the data's practicability and applicability will serve as merits for commercialization.

## 2 Background

Terpenes have attracted the attention of the industry, especially in cannabis-related industries. The combination of terpenes and cannabinoids produces a psychoactive effect (entourage effect) [1], which helps reduce anxiety [2], depression, and inflammation [3]. Infusion in pre-rolled cones is a convenient and popular method for consuming terpenes, and it offers a time-saving, consistent, portable, and customizable way to enjoy cannabis. Various terpene-infused pre-rolled cone products with various infusion and packaging methods have been marketed. Given their high volatility, terpene degradation in pre-rolled cones is the most challenging problem in product marketing. The terpene content in pre-rolled cones may be reduced when marketed. To date, no method has been reported for estimating the terpene content in infused cones, which makes determining terpene efficacy unclear. A simple and robust method for estimating terpene content in pre-rolled cones has been demonstrated here. This procedure was developed using the Salkowski test to detect terpenoids [[3], [4], [5]]. The terpenes in the rolled cones are extracted using chloroform and then precipitated using sulphuric acid. The terpene content was then estimated using a spectrophotometric procedure at a wavelength of 538 nm.

## 3 Data Description

**Fig. 1:**
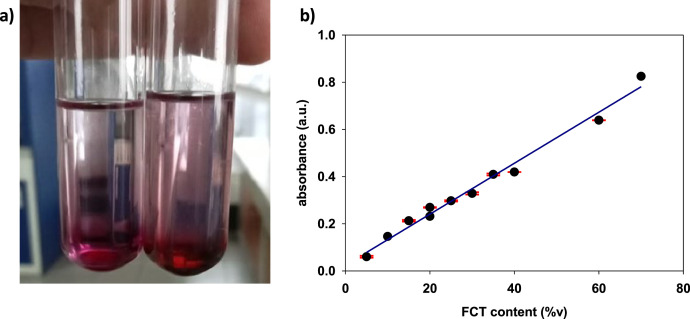
(a) Reddish brown precipitate formed from terpene in FCT. (b) Standard curve for determination of FCT content.

This paper shows the data on the amount of terpene loss from the infused pre-rolled cones by comparing the amount of FCT infused initially and after 7 days' storage. Estimation of FCT content was carried out based on the formed reddish brown precipitate after employing the measurement procedure by Ghorai et al. [6,7]; the formed precipitate is shown in Fig. 1a. The prepared standard calibration curve corresponding to the percent volume (%v) FCT is provided in Fig. 1b. The obtained data point in the standard curve are well-correlated to the linear model which follows eq. 1, with coefficient of determination (*R*^2^) value of 0.985.

(1) y=0.0108x+0.0245

where y is the absorbance at 538 nm, and x is the FCT content (%v). The obtained linear curve fitting equation is employed to estimate the FCT content in the infused cone paper.

### 3.1 Data on FCT decrement as the effect of tube sealing

**Fig. 2:**
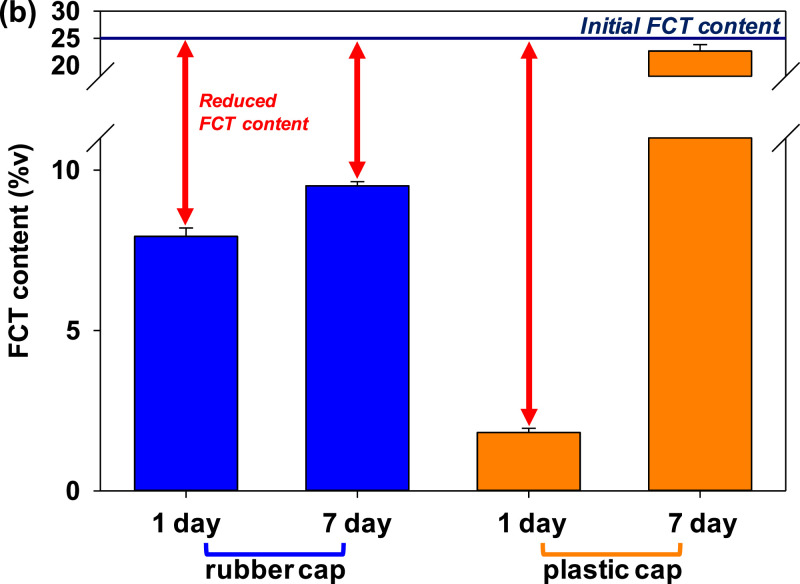
FCT content decrement as the effect of tube cap type (rubber cap vs. plastic cap) after 1 day and 7 days of storage.

The effect of using a plastic (polypropylene) screw cap and rubber cap on a glass tube on the FCT content after 7 days is shown in Fig. 2. The FCT content was reduced to 7.93 %v from initially 25 %v after 1 day stored, and the content was slightly increased to 9.51 %v. Meanwhile, 22.66 %v of FCT can be maintained after 7 days of being stored in a tube closed with a plastic cap. The use of rubber caps results in low FCT content after 7 days stored as the rubber absorbs the evaporated FCT; using plastic caps can dramatically reduce this issue.

### 3.2 Data on FCT decrement as the effect of added additives

**Fig. 3:**
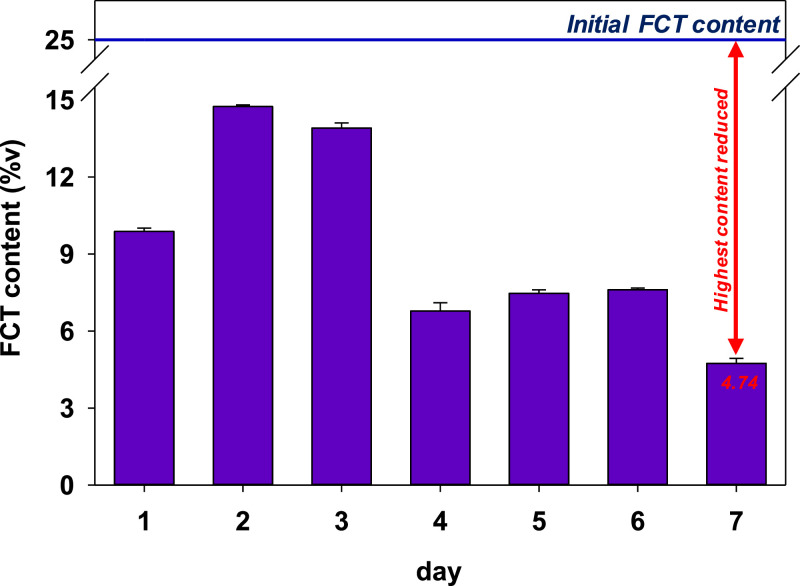
Detected FCT content after 7 days of incubation at room temperature. The final FCT content after 7 days of incubation is 4.74 %v.

Fig. 3 shows the reduced FCT content in cone paper after incubation for a certain period without any added additives. After 1 day of incubation, the FCT content was reduced to 9.88 %v from an initial content of 25 %v. The FCT was evaporated from the cone paper during the incubation, resulting in the loss of FCT content. Interestingly, a sharp increase of FCT content in the cone paper was detected after the second day of incubation, and the FCT content kept decreasing until the seventh day of incubation. Our findings highlighted an interesting phenomenon that can be correlated to the condensation of the evaporated FCT.

**Fig. 4:**
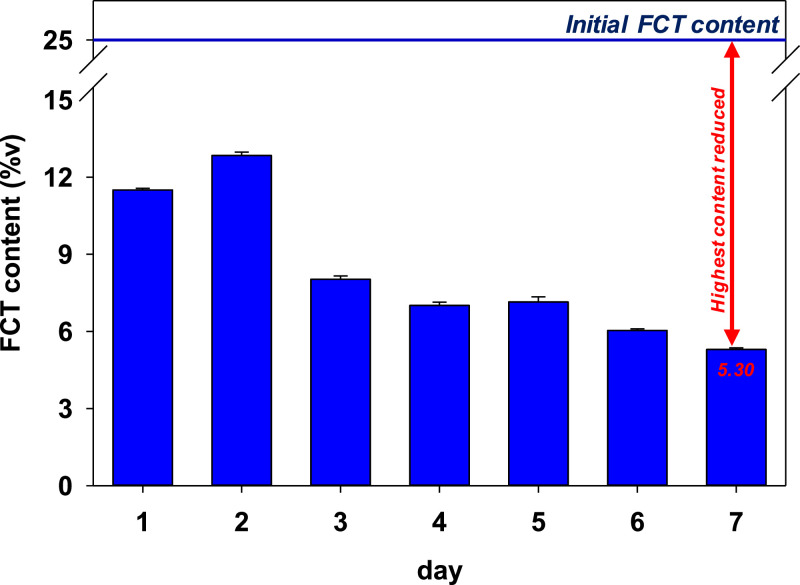
Detected FCT content after 7 days of incubation at room temperature with EtOH additive. The final FCT content after 7 days of incubation is 5.30 %v.

Terpene is soluble in ethanol (EtOH) [8]; thus, adding EtOH in infusing FCT may help enhance its absorption into the cone paper. The reduction of FCT content with the addition of EtOH is shown in Fig. 4. The FCT content was reduced after the first day of incubation. A similar phenomenon to the previous result was also observed, where the FCT content increased on the second incubation day. The FCT content was simultaneously decreased up to the seventh day of incubation.

**Fig. 5:**
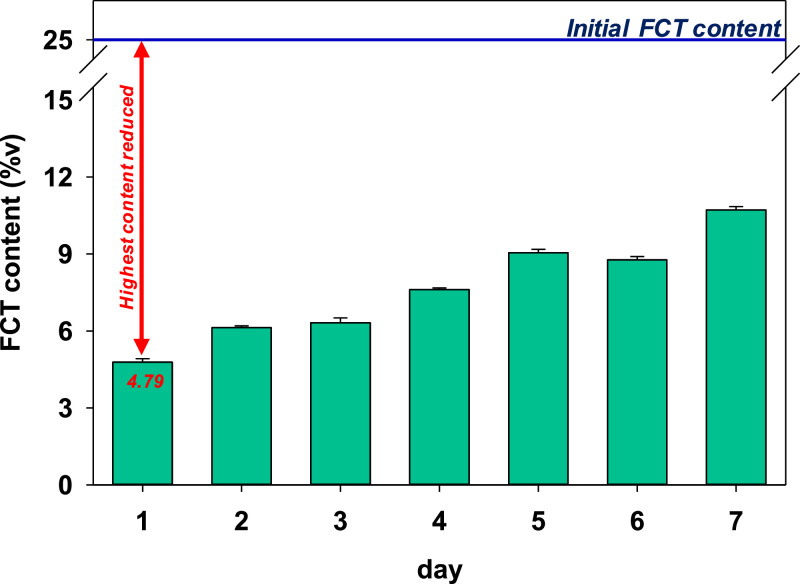
Detected FCT content after 7 days of incubation at room temperature with PPG additive. The final FCT content after 7 days of incubation is 10.71 %v.

The effect of polypropylene glycol (PPG) addition in the FCT content of the infused cone paper is shown in Fig. 5. It can be noted that a very high FCT content reduction occurred after the first day of incubation, which can be due to the effect of competition between the PPG and the FCT to be absorbed in the cone paper. Interestingly, the FCT content increased gradually at prolonged incubation time. This phenomenon could be correlated to the role of the absorbed PPG in aiding the fixating of the FCT into the cone paper. PPG is known to have adhesive properties [9]; thus, it adheres to the surface of cone paper after being heated during infusion.

**Fig. 6:**
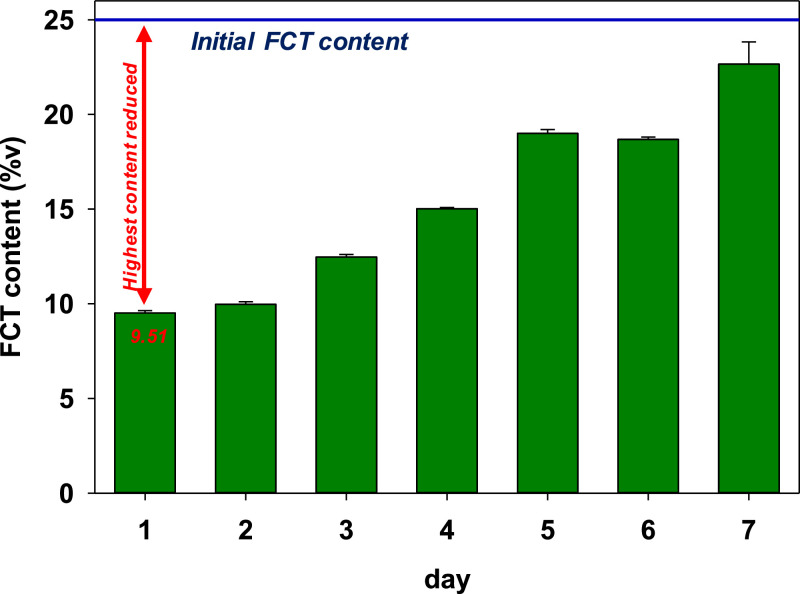
Detected FCT content after 7 days of incubation at room temperature, with EtOH and PPG additive. The final FCT content after 7 days of incubation is 22.65 %v.

Fig. 6 shows the effect of EtOH and PPG addition in decreasing FCT content during the incubation period. The combination of EtOH and PPG shows a synergistic effect in maintaining high FCT content after 7 days of incubation, with a final FCT content of 22.65 %v. This synergistic effect can be attributed to the role of EtOH in promoting the diffusion rate of both PPG and FCT into the cone paper and the role of PPG in preventing the saturation of the cone paper by moisture.

### 3.3 Data on FCT decrement from the infused glue

**Fig. 7:**
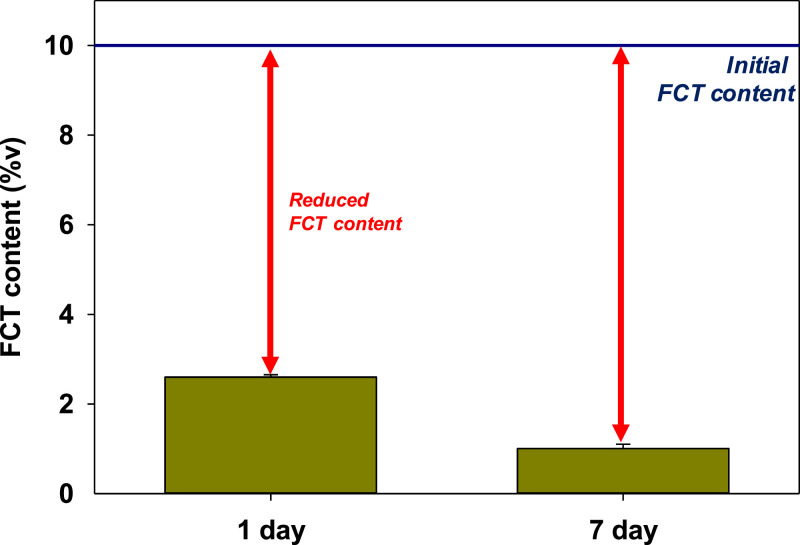
Detected FCT content in glue after 1 day and 7-day incubation.

Fig.7 shows the amount of FCT reduced from the glue mixture after being incubated for 1 day and 7 days. The FCT content was reduced from 10 %v to 2.60 %v after 1 day of incubation and further reduced to 1.01 %v after 7 days of incubation.

## 4 Experimental Design, Materials and Methods

### 4.1 Chemicals

FCT with 99 % purity was provided by Flow Scientific, Canada. Ethanol (EtOH, 96 % purity) was purchased from Supelco, MilliporeSigma. Chloroform (CHCl_3_, 99 % purity) and sulphuric acid (H_2_SO_4_, 98 % purity) were obtained from Honeywell, Indonesia. Polypropylene glycol (PPG, 99.8 % purity) was obtained from Dow Chemical, Thailand.

The specifications of the pre-rolled cone papers used in this paper are detailed in Table 1.

**Table 1:** Specification of pre-rolled cone paper.

Parameter	Unit	Target	Min	Max	Test method
Grammage	g/m^2^	14.0	13.0	15.0	ISO 536
Thickness (1 layer)	µm	27.0	Typical value		ISO 534
Tensile strength	N/15 mm	16.0	13.0	-	ISO 1924-2
Stretch at break	%	1.3	1.1	-	ISO 1924-2
Opacity	%	48.0	-	52.0	ISO 2471
Brightness ISO	%	20.0	15.0	-	ISO 2470
Air permeability	CU	5	-	15.0	ISO 2965
Fiber furnish	Wood				

Testing conditions: 50 % relative humidity, 23 °C

All of the chemicals and apparatus used in this study were the same as those intended for large scale production.

### 4.2 FCT standard curve preparation

The FCT standard curve was prepared by adopting the method of Ghorai et al. (2012), with slight modifications. The FCT was pipetted into a reaction tube at various amounts, as shown in Table 2. Then, ethanol was added to make up the total volume of 200 µL. Subsequently, 1.5 mL of chloroform was added, and the sample was vortexed for 3 min. 100 µL of H_2_SO_4_ was added, and the sample was incubated for 1.5 h in the dark. After this step, a reddish brown precipitation was formed; the precipitation was partially soluble in the mixture. The supernatant was gently decanted without disturbing the precipitation. Then, 2.8 mL of methanol was added to dissolve the precipitation. The absorbance of the dissolved sample was then measured using a UV-Vis spectrophotometer (Genesys 150, Thermo Fisher Scientific, USA) at 538 nm.

**Table 2:** Concentration of FCT expressed in percent volume.

Vol. of FCT (µL)	Vol. of EtOH (µL)	FCT Content (%v)*
140	60	70
120	80	60
100	100	50
80	120	40
60	140	30
40	160	20

⁎ $\text{FCT}\;\text{content}\;\left(\%v\right)=\frac{\text{Vol}.\;\text{of}\;\text{FCT}}{\text{Vol}.\;\text{total}}\times 100$.

### 4.3 Method for infusing FCT into pre-rolled cones

The infusion of FCT into the pre-rolled cone is carried out following the standard production protocol of the relevant product that applies at Mitra Prodin, at which the data will be mostly applied. Briefly, pre-rolled cone paper was put into a screw-capped borosilicate test tube with a 25 × 150 mm dimension. The screw cap was made of polypropylene. 50 µL of FCT was injected into the terpene storage attached to a tube cap. Then, the tube was closed and heated at 80 °C in an oven (UN55, Memmert, Germany) for 60 min.

In this study, the effect of EtOH and PPG addition was also evaluated. Infusion tests were carried out using the following formulations: FCT only, FCT + EtOH (volume ratio 1:1), FCT+PPG (ratio 1:1), and FCT + EtOH + PPG (ratio 1:1:1).

### 4.4 Method for mixing FCT and dextrin glue

The mixing of FCT and the dextrin glue is carried out following the standard production protocol of the relevant product that applies at PT Mitra Prodin. Dextrin-based glue was mixed with FCT and PPG, with the composition shown in Table 3.

**Table 3:** Chemical composition of terpene-base glue.

Composition	% weight
Dextrin-based glue	85 %
Terpene	10 %
Polypropylene Glycol	5 %

PPG and FCT were first vigorously mixed using a vortex mixer for 3 min. Then, the mixture was added to dextrin-based glue at the designated ratio. The mixture was then homogenized using a vortex mixer for 5 min.

### 4.5 Method on the estimation of FCT content

The FCT-infused cone paper was put in a 20 mL capped glass tube containing 1.5 mL chloroform. The cone paper was pressed and vortexed for 3 min, then the residue was removed. 100 µL of H_2_SO_4_ was added into the solution of FCT in chloroform, and the mixture was incubated for 1 h at room temperature with dark conditions. After incubation, the reddish-brown precipitation was formed, and the clear upper layer solution was gently decanted. Subsequently, 2.8 mL of methanol was added to dissolve the precipitation. The absorbance of the sample was measured with a wavelength of 538 nm to determine the FCT content.

## Limitations

This study is subjected for practical purposes in estimating the terpene content in the form of FCT. All of the terpene measurements were expressed as the percent volume of FCT.

## Ethics Statement

The data in this work does not involve human subjects, animal experiments, or any data collected from social media platforms.

## CRediT authorship contribution statement

**Arthur Karangan:** Conceptualization, Methodology, Investigation, Formal analysis, Data curation, Writing – original draft. **Sherwin Wardhana Rahardjo:** Conceptualization, Supervision. **Antonius Jimmy Widagdo:** Formal analysis, Visualization. **Shella Permatasari Santoso:** Supervision, Writing – review & editing. **Suryadi Ismadji:** Supervision, Writing – review & editing.

## Data Availability

Data on terpene content in pre-rolled paper cones infused with terpene-containing flavours based on the formation of a partially soluble precipitate (Original data) (Mendeley Data).
